# An algorithm of discovering signatures from DNA databases on a computer cluster

**DOI:** 10.1186/1471-2105-15-339

**Published:** 2014-10-05

**Authors:** Hsiao Ping Lee, Tzu-Fang Sheu

**Affiliations:** Department of Medical Informatics, Chung Shan Medical University, 110, Sec. 1, Jianguo N. Road, 40201 Taichung, Taiwan; Department of Medical Research, Chung Shan Medical University Hospital, 110, Sec. 1, Jianguo N. Road, 40201 Taichung, Taiwan; Department of Computer Science and Communication Engineering, Providence University, 200, Sec. 7, Taiwan Boulevard, 43301 Shalu Dist, Taichung, Taiwan

**Keywords:** Signature discovery, Computer clusters, Divide-and-conquer strategies

## Abstract

**Background:**

Signatures are short sequences that are unique and not similar to any other sequence in a database that can be used as the basis to identify different species. Even though several signature discovery algorithms have been proposed in the past, these algorithms require the entirety of databases to be loaded in the memory, thus restricting the amount of data that they can process. It makes those algorithms unable to process databases with large amounts of data. Also, those algorithms use sequential models and have slower discovery speeds, meaning that the efficiency can be improved.

**Results:**

In this research, we are debuting the utilization of a divide-and-conquer strategy in signature discovery and have proposed a parallel signature discovery algorithm on a computer cluster. The algorithm applies the divide-and-conquer strategy to solve the problem posed to the existing algorithms where they are unable to process large databases and uses a parallel computing mechanism to effectively improve the efficiency of signature discovery. Even when run with just the memory of regular personal computers, the algorithm can still process large databases such as the human whole-genome EST database which were previously unable to be processed by the existing algorithms.

**Conclusions:**

The algorithm proposed in this research is not limited by the amount of usable memory and can rapidly find signatures in large databases, making it useful in applications such as Next Generation Sequencing and other large database analysis and processing. The implementation of the proposed algorithm is available athttp://www.cs.pu.edu.tw/~fang/DDCSDPrograms/DDCSD.htm.

## Background

Mutations give diversity to DNA sequences, which led to the evolution of a variety of different species and a multitude of species from the same ancestor. Even though they have similar DNA sequences from a common ancestor, due to evolution, these species also have their own unique DNA sequences which may be understood as the signatures for those particular species and can be used as a way to separate the species[1, 2]. For example, DNA signatures have already been used to identify 14 types of human pathogenic yeast[3].

Signatures are defined as DNA patterns that are significantly different from other sequences and appear only once in the sequence database. Thus, the purpose of signature discovery is to find all of the signatures in a database[4]. Much research has already been conducted in signature discovery. Amin et al. integrated multiple bioinformatics tools, including CG[5] and IslandPath[6], to determine horizontally transferred, pathotype-specific signature genes as targets for specific, high-throughput molecular diagnostic tools and reverse vaccinology screens[7]. PrimerHunter can be used to select highly sensitive and specific primers for virus subtyping identification[8]. To guarantee high sensitivity and specificity, PrimerHunter selects primers such that they efficiently amplify one of the target sequences representing different isolates of the subtype of interest, and none of the non-target sequences representing isolates of closely related virus subtypes. Accurate estimates of the melting temperature of mismatches, based on a nearest-neighbor model and calculated via a fractional programming algorithm, are used in PrimerHunter to ensure the desired amplification properties. TOFI is a tool for identifying oligonucleotide fingerprints for microarray-based pathogen diagnostic assays, which combines genome comparison tools, probe design software, and sequence alignment programs[9, 10]. TOFI is typically used to design fingerprints for a single genome. An enhanced multiple-genome pipeline presented by Satya et al. allows for efficient design of microarray probes common to groups of target genomes[11]. Insignia is web-based tool for identifying genomic signatures that are perfectly conserved by all target genomes and absent from all background genomes based on databases of bacterial and viral genomic sequences, which comprise over 8300 distinct organisms[12, 13]. TOFI designs signatures for microarray-based assays, and Insignia finds unique sequence segments that can be used to design both PCR and microarray signatures. Insignia and TOFI have the ability to identify genomic signatures that are common to multiple target genomes. Insignia and TOFI perform similar computations, but Insignia can be run online and requires less computational resources. TOFI and Insignia both build consensus regions among multiple genomes through pairwise alignments between the target genomes. Insignia reports only the unique segments in the target genomes and provides an option for users to run Primer3[14], a PCR signature design software, on these unique segments. To quickly identify signatures in target and background genomes, Insignia has to maintain a specialized database containing pre-computed matches between every pair of genomes. However, the concomitant advantage in speed comes with the limitation that users are restricted to the target and background genomes that are part of the Insignia database, with no option to use other sequences as target or background genomes. TOPSI is a tool that extends the TOFI framework to design signatures for PCR-based pathogen diagnostic assays[15]. Like Insignia, TOPSI identifies unique segments through pairwise alignments between the input genomes. However, TOPSI goes beyond identification of unique segments, and incorporates modules to design PCR signatures from the unique segments and perform extensive specificity analysis on the designed signatures. TOPSI can provide a list of PCR signatures common to all input targets without manual manipulation. CaSSiS is capable of computing comprehensive sets of sequence- and group-specific signatures that guarantee a predefined Hamming distance, the number of mismatches with non-target sequences, from collections of deeply hierarchically clustered sequences[16]. CaSSiS tries to determine perfect group-covering signatures for every target group. For groups lacking a perfect common signature, CaSSiS finds signatures with maximal group coverage within a user-defined specificity. Zheng’s algorithm uses the Hamming distance between sequences as a measuring stick for signature discovery[17]. Suppose *l* and *d* are two whole numbers. An *l*-pattern represents the DNA sequence with a length of *l*. If two *l*-patterns are (*l,d*)-similar, this means that the Hamming distance between the two *l*-patterns does not exceed *d*. Moreover, if (*l,d*)-similar *l*-patterns could not be found, the pattern is defined as a signature under the discovery condition (*l,d*) in the database. Zheng’s algorithm can find all of the signatures in a database as defined above. The IMUS algorithm improved upon Zheng’s algorithm to give better discovery efficiency, but requires a larger memory[18]. Based on mathematical analysis, if a discovery condition is set as (*l* = 24,*d* = 4), when discovering signatures in a uniformly distributed database with a size of 2^30^, IMUS requires only 7.4% of the string comparisons made by Zheng’s algorithm but creates 256 times more entries in the index. CMD is designed to discover all implicit signatures from DNA databases, where implicit signatures are signatures that satisfy discovery conditions looser than a given discovery condition[19].

However, none of the above algorithms distribute the computation of the databases onto multiple computers in a cluster. To use the algorithms in such a way, additional scripts must be applied to control the distribution and collection of the databases and results. Unfortunately, many of these approaches do not provide a formal definition for their distribution strategies. Some of the approaches, for example Insignia and CaSSiS, provide strategies to distribute the computation of the databases onto multiple computers in a cluster, but the steps of distribution and collection are not automatic. Manual manipulation is necessary to use these algorithms to distribute the computation of the databases onto multiple computers in a cluster. The match pipeline in Insignia applies strategies to reduce redundancy in sub-datasets, but relies mainly on preprocessing. PTPan[20], Jellyfish[21] and DSK[22] apply different strategies to avoid the necessity of loading the whole database into memory for searches. Each of the three approaches uses secondary storage. For example, Jellyfish and DSK use hash tables to compute the *k*-mers for a given *k*. Both algorithms achieve space efficiency by keeping most of the hash tables on disk. When counting *k*-mers over multiple hash tables, Jellyfish would need to store the intermediate *k*-mer counts on disk, which requires significantly more space, and the merge phase is not parallelized. This makes the algorithm time intensive for large databases. IMUS and Zheng’s algorithm both have two disadvantages. First, these algorithms require that the entire database to be processed (including all of the data structures that were used during computation) be loaded into memory, meaning that when the amount of data exceeds the memory capacity, these algorithms are unable to complete processing and cannot be used. Second, they are both sequential algorithms, so the time necessary for larger databases is extensive. Due to these two disadvantages, neither IMUS nor Zheng’s algorithm is suitable for applications that require processing large databases. This is a particular problem with the development of Next Generation Sequencing (NGS), as the rate of creation of sequence data is increasing daily, leading also to larger databases. This renders both IMUS and Zheng’s algorithm, which are unable to process large amounts of data and require longer processing times, unsuitable for NGS data analysis.

Divide-and-conquer is a computational strategy for solving both extensive and complicated problems and processing large amounts of data. The basic thought behind this is as follows: suppose the amount of data that needs to be processed for a problem is represented by |*D*|. If |*D*| is smaller, it can be easily solved and can be solved directly. Otherwise, the problem may be divided into multiple smaller scale subproblems with close similarities to the original problem. These subproblems may be solved recursively, and the results combined to find a solution to the original problem. Therefore, with the divide-and-conquer strategy, each recursion may include three main steps: (1) solve: if the problem is smaller in scale and easy to solve, it looks for a solution directly; (2) divide-and-recur: divide the original problem into multiple smaller scale subproblems closely similar to the original problem, then recursively try to find the solution to each subproblem; (3) combine: take the solutions from the subproblems and combine to find the solution to the original problem[23]. In addition, as technology has matured, the price of multi-core CPUs has continued to fall, so the possibility to use parallel processing technology on a computer cluster to enhance processing efficiency has greatly improved. In fact, parallel processing technology is already used in many bioinformatics research fields, such as sequence alignment and analysis, protein structure prediction, and motif finding[24–31]. If we can use the divide-and-conquer strategy and parallel processing technology in signature discovery, this will improve the efficiency of discovery in large databases, which will be immensely helpful.

In this research, we propose a signature discovery algorithm called distributed divide-and-conquer-based signature discovery (DDCSD) algorithm. The DDCSD algorithm is designed specifically for discovering signatures on a computer cluster. The DDCSD algorithm automatizes the steps of distributing the database and collecting the unique signatures. The signatures are discovered from the database and provided to users without manual manipulation. The DDCSD algorithm uses the divide-and-conquer strategy to overcome the problem of processing large databases and compares multiple patterns in parallel to accelerate signature discovery. Therefore, the algorithm not only shortens the amount of time needed for discovery, it also is able to process the large databases that could not be processed in the past using IMUS and Zheng’s algorithm. In addition, by setting the threshold value of the direct discovery, DDCSD can limit the memory requirement in discovery to the memory size of the computers in the cluster. More specifically, the DDCSD algorithm can process any amount of data and is not limited by the amount of memory available. The DDCSD algorithm is implemented using a basic divide-and-conquer strategy as the basic structure. First, it decides whether to do direct discovery based on the size of the database. If the database is too large to load in its entirety, it will split the database into two equal parts and recursively processes the parts. As the recursive processing is in progress, the amount of data in a single part will gradually decrease until it can load the single part all into the memory of one computer in the cluster at one time. At the end, it will combine the results that were found separately in the two different parts and find the signatures in the original database. The DDCSD algorithm gives the formal definition in recursion for the dataset distribution strategy, that is not provided by the previous approaches. The DDCSD algorithm includes main and discovery routines. The main routine organizes discovery in a planned way. The discovery routine is used to find the unique patterns from a specified dataset in another dataset. The computation of discovery and collection in DDCSD is distributed onto discovery nodes for parallelization. Based on the experiments made on the human whole-genome EST database that has approximately 2.46G bases, the DDCSD algorithm proposed here can successfully process that database. Whereas previous algorithms could not process databases so large, the DDCSD algorithm took 1.89 hours to find all of the signatures under the discovery condition (30,2) on the cluster of ten discovery computers with 32 GB memory. The main contribution of this research is utilizing the divide-and-conquer strategy in signature discovery to process discovery in large databases, something previous algorithms were unable to do, and providing a parallel signature discovery algorithm on a cluster, that can process databases of any size regardless of the amount of memory available. This algorithm can be applied to NGS data analysis and other analysis of large databases.

## Methods

Suppose that *l* and *d* represent the length and the number of allowed mismatches of signatures, respectively, and Λ is a dataset made up of *l*-patterns. We define signatures in Λ under a discovery condition (*l*,*d*) as patterns that exist in Λ and where there are no other (*l,d*)-similar patterns inside of Λ. The purpose of this research is to utilize a divide-and-conquer strategy to provide a parallel algorithm that can rapidly discover the signatures in datasets with massive amounts of data on a computer cluster.

For any subset Θ of Λ, if no (*l,d*)-similar pattern can be found in Θ, this pattern is considered unique in Θ. According to this definition, we can deduce that if one pattern *P* is a signature in Λ, then *P* must be unique in Θ. Therefore, if we divide Λ into two partitions of equal size (Λ_*i*_ and Λ_*j*_), then *P* will be a signature for either Λ_*i*_ or Λ_*j*_ and will be unique to the other partition. Thus, when the signatures of Λ_*i*_ and Λ_*j*_ are combined, they will include all of the signatures for Λ, making them valid candidates to discover signatures in Λ. Most importantly, no matter how many levels of recursive processing are applied, this characteristic still stands, meaning that we can use the divide-and-conquer strategy on a computer cluster to deal with the original problem posed to signature discovery algorithms where they could not process large databases. Using the above as the foundation, we designed a distributed divide-and-conquer-based signature discovery (DDCSD) algorithm that can rapidly discover the signatures that satisfy the discovery condition (*l*,*d*) in a large dataset on a computer cluster. The DDCSD algorithm includes main and discovery routines. The discovery routine accepts the candidate and source datasets that are made up of *l*-patterns and will find the patterns that are unique in the source in the candidate. It must be made clear that when the candidate and source are set as the same dataset, the patterns found by the discovery routine are the signatures for the dataset. Each of the computers in the cluster is called a node. The node that handles the main routine is called a main node, and those that handle the discovery routine are called discovery nodes.

**Figure 1:**
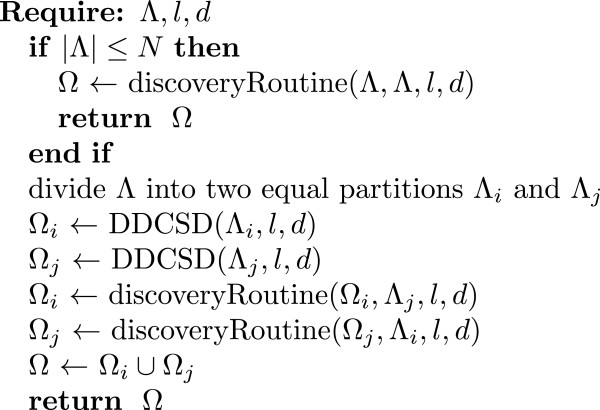
**The main routine of the DDCSD algorithm.** The algorithm discovers signatures from Λ under the discovery condition (*l*,*d*), where *l* and *d* are the length and the number of allowed mismatches of the signatures, and, Λ is a dataset made up of *l*-patterns. |Λ| represents the number of patterns in Λ. *N* is the preset threshold value for direct discovery.

The main routine of the DDCSD algorithm is shown in Figure1. The symbols used in DDCSD are presented in Table1. The DDCSD algorithm first examines the size of the dataset Λ; if the number of patterns is less than or equal to the preset threshold value, *N*, then it will automatically send Λ to a discovery node and use the discovery routine to find the signatures in Λ. The discovery result is sent back to the main node. The threshold value is decided based on the memory space of discovery nodes and is set so that the patterns in Λ (including all of the used data structures) can be loaded into the memory. On the other hand, if the number of patterns is more than the preset threshold, then Λ will be divided into two equal partitions Λ_*i*_ and Λ_*j*_, with each being recursively processed individually. After recursive processing, the algorithm combines the results to find the signatures of Λ. Suppose that Ω_*i*_ and Ω_*j*_ represent all of the signatures in Λ_*i*_ and Λ_*j*_, respectively. Because all signatures of Λ must be present in either Ω_*i*_ or Ω_*j*_ and will be unique to the other partition, additional comparisons must be made between Ω_*i*_ and Λ_*j*_ as well as between Ω_*j*_ and Λ_*i*_ to find the patterns unique in the other partition and delete the non-unique ones. Therefore, DDCSD uses the discovery routine to find the unique patterns in Λ_*j*_ that are in Ω_*i*_ and the unique patterns in Λ_*i*_ that are in Ω_*j*_. These two discovery results are then combined, with the final result being the signatures of Λ.

**Table 1:** **The symbols and their definitions in the DDCSD algorithm**

Symbols	Definitions
*l*	The length of signatures
*d*	The number of allowed mismatches of signatures
*N*	The threshold value of direct discovery
Λ	The input dataset made up of *l*-patterns
Λ_*k*_	A partition of Λ, *k* = 1,2,…
Ω	The set of signatures in Λ
Ω_*k*_	The set of signatures in Λ_*k*_
Θ	The source in the discovery routine, corresponding to
	Λ or Λ_*k*_ in the main routine
Ξ	The candidate in the discovery routine, corresponding to
	Ω or Ω_*k*_ in the main routine

The following example illustrates the processes of DDCSD. It is assumed that the number of patterns in a given dataset Λ is |Λ| = 4*N*, where *N* is the threshold value of direct discovery. As |Λ| is more than *N*, according to the processing rule of DDCSD, Λ is divided into two partitions, Λ_1_ and Λ_2_, of equal size with |Λ|/2 = 2*N*. As |Λ_1_| is still greater than *N*, Λ_1_ is further divided into two partitions, Λ_3_ and Λ_4_, of size *N*. Now, since |Λ_3_| is not greater than *N*, DDCSD stops dividing Λ_3_ into partitions, and executes DiscoveryRoutine (Λ_3_,Λ_3_,*l*,*d*) on a discovery node, which yields the signature set, Ω_3_, from Λ_3_. Similarly, as |Λ_4_| is not more than *N*, DDCSD directly discovers the signature set Ω_4_ from Λ_4_ on a discovery node. Then, DDCSD executes DiscoveryRoutine (Ω_3_,Λ_4_,*l*,*d*) and DiscoveryRoutine (Ω_4_,Λ_3_,*l*,*d*) on discovery nodes, respectively. The union of the obtained Ω_3_ and Ω_4_ is the signature set, Ω_1_, of Λ_1_. Similarly, DDCSD processes Λ_2_ by dividing it into two partitions, Λ_5_ and Λ_6_, each with a size of *N*. From Λ_5_ and Λ_6_, the signature sets Ω_5_ and Ω_6_ are discovered. Then, Ω_5_ and Ω_6_ are combined to obtain the signature set of Λ_2_, namely Ω_2_. Finally, DDCSD combines Ω_1_ and Ω_2_ to get the signature set of Λ, namely Ω. Table2 shows the sequence of signature sets identified by DDCSD. The required processes for discovering the signature sets are also presented in the table. The discovery processes are encoded for clear illustration. Table3 lists the processing time of the discovery processes shown in Table2. Assume that *A*, *B*, *C*, *D*, *E*, *F*, *G*, *H*, *I* and *J* are discovery processes. The cluster contains two discovery nodes, namely *DN*_1_ and *DN*_2_. First, DDCSD assigns *DN*_1_ and *DN*_2_ to discovery processes *A* and *B*, respectively. After the processing of discovery process *A* is completed, *DN*_1_ is immediately assigned to the next discovery process, discovery process *C*. *DN*_1_ and *DN*_2_ are assigned to process the discovery processes, until all of the discovery processes are completed. In this case, *DN*_1_ is assigned to discovery processes *A*, *C*, *E*, *F*, *G* and *J*, and *DN*_2_ is assigned to discovery processes *B*, *D*, *H* and *I*. The discovery time consumed by *DN*_1_ and *DN*_2_ is 15 seconds each, so the overall discovery time is 15 seconds. It is noteworthy that some of the discovery processes are sequentially interdependent. The sequential interdependence might affect the overall processing time for discovering signatures on the computer cluster. That is, when the preceding discovery process needs relatively more processing time, the successive dependent discovery process might have to wait. For example, DiscoveryRoutine (Ω_6_,Λ_5_,*l*,*d*) can only be executed after DiscoveryRoutine (Λ_6_,Λ_6_,*l*,*d*) is completed, that is, discovery process *H* can only be executed after discovery process *F* is done. Assume the processing time of discovery process *F* is 7 seconds. In this case, although discovery process *G* is completed, discovery process *H* cannot be immediately processed because discovery process *F* is still executing. Therefore, discovery process *H* has to wait. This reduces the discovery efficiency of DDCSD.

**Table 2:** **An example of DDCSD**

Order	Candidate	Process	Process ID
1	Ω_3_	DR(Λ_3_,Λ_3_,*l*,*d*)	*A*
2	Ω_4_	DR(Λ_4_,Λ_4_,*l*,*d*)	*B*
3	Ω_1_	DR(Ω_3_,Λ_4_,*l*,*d*)	*C*
		DR(Ω_4_,Λ_3_,*l*,*d*)	*D*
4	Ω_5_	DR(Λ_5_,Λ_5_,*l*,*d*)	*E*
5	Ω_6_	DR(Λ_6_,Λ_6_,*l*,*d*)	*F*
6	Ω_2_	DR(Ω_5_,Λ_6_,*l*,*d*)	*G*
		DR(Ω_6_,Λ_5_,*l*,*d*)	*H*
7	Ω	DR(Ω_1_,Λ_2_,*l*,*d*)	*I*
		DR(Ω_2_,Λ_1_,*l*,*d*)	*J*

The Order field presents the order of the signature sets identified by DDCSD. The Process field presents the required process for discovering each signature set. The Process ID field lists the represented code of each discovery process. DR() is the abbreviation of DiscoveryRoutine().

**Table 3:** **Processing time for the discovery processes shown in Table** 2

Process ID	***A***	***B***	***C***	***D***	***E***	***F***	***G***	***H***	***I***	***J***
Time	1	3	4	6	2	1	4	2	4	3

The Process ID field lists the discovery processes shown in Table2. The Time field presents the processing time of each discovery process. The time unit is seconds.

Suppose that *P* and *Q* are two *l*-patterns. If *P* is divided into equal and non-overlapping ⌈*l*/*γ*⌉ number of *γ*-patterns, these *γ*-patterns are called *γ*-segments of *P*. *P*_*γ*,*i*_ represents the *i*-th *γ*-segment in *P*. *P* is called (*γ*,*i*,*δ*)-matched to a *γ*-pattern Γ if *P*_*γ*,*i*_ is (*γ*,*δ*)-similar to Γ. We arrive at the observation that if *P* and *Q* are (*l*,*d*)-similar, for a given *γ*, there will be at least one *i* such that *P* is (*γ*,*i*,⌊*γd*/*l*⌋)-matched to *Q*_*γ*,*i*_. Using the observation as the foundation, we designed the discovery routine of DDCSD.

**Figure 2:**
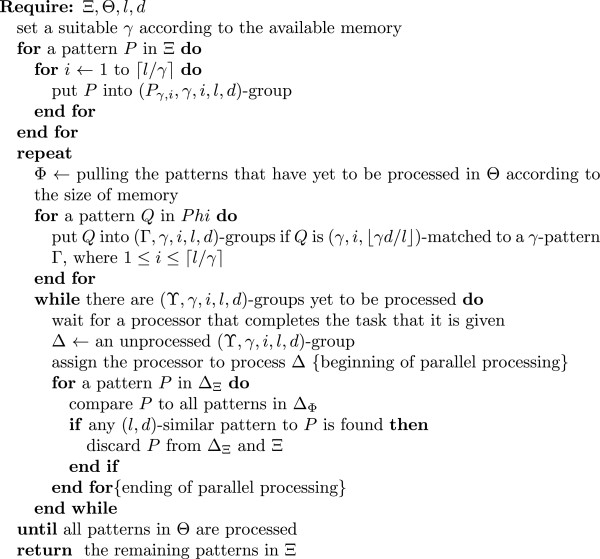
**The discovery routine of the DDCSD algorithm.** The algorithm runs on a discovery node. The algorithm discovers the unique patterns from Θ that are in Ξ under the discovery condition (*l*,*d*), where *l* and *d* are the length and the number of allowed mismatches of signatures, and, Ξ and Θ are datasets made up of *l*-patterns.

The discovery routine of the DDCSD algorithm runs on discovery nodes. The discovery routine allows multiple processors to compare similarity of different patterns at the same time to allow for faster discovery speed. The discovery routine is shown in Figure2. Suppose that the candidate and source datasets received by the discovery routine are Ξ and Θ, respectively. First of all, the discovery routine will set a suitable *γ* according to the memory that is available in the discovery node, where *γ* is a whole number between ⌈*l*/(*d*+1)⌉ and ⌈*l*/2⌉. The larger the number, the less strings are compared during a discovery, but more memory is needed. Conversely, the smaller the number, the more strings are compared during a discovery, but the memory requirement will be smaller. Suppose that *Υ* is a *γ*-pattern. A (*Υ*,*γ*,*i*,*l*,*d*)-group is a group of *l*-patterns. All of the *l*-patterns are (*γ*,*i*,⌊*γd*/*l*⌋)-matched to *Υ*. According to the *γ*-segments included in the *l*-patterns in Ξ and Θ, the discovery routine assigns the *l*-patterns to (*Υ*,*γ*,*i*,*l*,*d*)-groups. More specifically, if *P* is in Ξ, then *P* will be put into (*P*_*γ*,*i*_,*γ*,*i*,*l*,*d*)-groups, where 1 ≤ *i* ≤ ⌈*l*/*γ*⌉. For exampmle, assume that *l*, *d* and *γ* are 4, 2 and 2, respectively. If *P* = ’ACGT’, then *P* will be put into (’AC’,2,1,4,2)- and (’GT’,2,2,4,2)-groups. Assume that Δ is one of the (*P*_*γ*,*i*_,*γ*,*i*,*l*,*d*)-groups. Δ_Ξ_ represents the set of *P*s in Δ. According to the size of the memory, the discovery routine can pull the patterns that have yet to be processed in Θ. If there are too many patterns in Θ which cannot be loaded into the memory all at once, it can split them into multiple parts and load and process the parts one at a time. Suppose that Φ represents the group of patterns that are loaded at this time. If *Q* is in Φ and *Q* is (*γ*,*i*,⌊*γd*/*l*⌋)-matched to a *γ*-pattern Γ, *Q* will be put into (Γ,*γ*,*i*,*l*,*d*)-groups, where 1 ≤ *i* ≤ ⌈*l*/*γ*⌉. For example, assume that *l*, *d* and *γ* are 4, 2 and 2, respectively. If *Q* = ’TGCA’, then *Q* will be put into (Γ_1_,2,1,4,2)- and (Γ_2_,2,2,4,2)-groups, where Γ_1_ ∈ {’TG’,’GG’,’CG’,’AG’,’TT’,’TC’,’TA’} and Γ_2_ ∈ {’CA’,’AA’,’GA’,’TA’,’CC’,’CG’,’CT’}. Assume that Δ is one of the (Γ,*γ*,*i*,*l*,*d*)-groups. Δ_Φ_ represents the set of *Q*s in Δ. Pairing this definition with the previous observation, we find that the patterns that are (*l, d*)-similar to the patterns in Δ_Ξ_ must be present in Δ_Φ_. Therefore, for the patterns in Δ_Ξ_, when examining whether they are unique, this principle can be applied to limit the discovery to similar patterns to those patterns to decrease the number of patterns compared. Each time a processor in the discovery node completes the task that it is given, the discovery routine takes a (*Υ*,*γ*,*i*,*l*,*d*)-group for that processor to process, which allows for parallel processing. Suppose the (*Υ*,*γ*,*i*,*l*,*d*)-group taken was Δ. For an *l*-pattern *P* in Δ_Ξ_, when searching for (*l*,*d*)-similar patterns to *P* in Φ, the discovery routine only compares *P* and the patterns in Δ_Φ_ to find whether there are (*l*,*d*)-similar patterns to *P*. If no (*l*,*d*)-similar pattern to *P* is found in Δ_Φ_, then it means that *P* is unique in Φ. Conversely, it is not unique and is deleted. The discovery routine repeats the above process until all (*Υ*,*γ*,*i*,*l*,*d*)-groups are processed.

## Results and discussion

### Mathematical analysis

The time complexity of the discovery routine used in the DDCSD algorithm is analyzed and the results are integrated, yielding the time complexity of the DDCSD algorithm. The memory consumption is also analyzed.

Suppose that *l* and *d* represent the length and the number of allowed mismatches of signatures, respectively. *γ* is a whole number between ⌈*l*/(*d* + 1)⌉ and ⌈*l*/2⌉. Ξ and Θ that are made up of *l*-patterns are the candidate and source datasets received by the discovery routine. |Ξ| and |Θ| denote the number of patterns in Ξ and Θ. According to the *γ*-segments included in the *l*-patterns in Ξ and Θ, the *l*-patterns are assigned to (*Υ*,*γ*,*i*,*l*,*d*)-groups, where *Υ* is a *γ*-pattern and 1 ≤ *i* ≤ ⌈*l*/*γ*⌉. Assume Ψ is the set of all possible (*Υ*,*γ*,*i*,*l*,*d*)-groups. Δ ∈ Ψ. Δ_Ξ_ = Δ ∩ Ξ and Δ_Θ_ = Δ ∩ Θ. Since the patterns that are (*l*,*d*)-similar to the patterns in Δ_Ξ_ must be in Δ_Θ_, each pattern in Δ_Ξ_ requires |Δ_Θ_| string comparisons to check whether it is unique, where |Δ_Ξ_| and |Δ_Θ_| are the number of patterns in Δ_Ξ_ and Δ_Θ_, respectively. Each of the string comparisons includes *α* = *l*-*γ* character comparisons. The total number of character comparisons in the discovery routine, denoted as *T*_*DR*_(|Ξ|,|Θ|), is:

Suppose that Ξ and Θ are in uniform distribution. In this case, Ψ should contain 4^*γ*^*β* (*Υ*,*γ*,*i*,*l*,*d*)-groups, where *β* = ⌈*l*/*γ*⌉. |Δ_Ξ_| ≈ *β*|Ξ|/(4^*γ*^*β*) = |Ξ|/4^*γ*^ and |Δ_Θ_| ≈ *κβ*|Θ|/(4^*γ*^*β*) = *κ*|Θ|/4^*γ*^, where. In the uniformly distributed case, the total amount of character comparisons in the discovery routine, denoted as, is:

Suppose that the input dataset Λ has a uniform distribution, and contains |Λ| = 2^*n*^*N* patterns, where *N* is the threshold value for direct discovery and *n* is a whole number. In each recursion, the division can be done by performing a sequential scan on Λ when dividing Λ into two partitions. The computational cost of division is *η*_1_|Λ|, where *η*_1_ is a constant. The amount of patterns sent to and received from discovery nodes in data transmission for processing each partition are all |Λ|/2. The total computational cost for data division and transmission in each recursion is *η*_1_|Λ| + 2(*η*_2_|Λ|/2 + *η*_3_|Λ|/2) = *η*_0_|Λ|, where *η*_0_, *η*_2_ and *η*_3_ are constants and *η*_0_ = *η*_1_ + *η*_2_ + *η*_3_. The computational cost of using DDCSD to discover signatures from Λ, denoted as, is:

where and *η* = *nη*_0_.

The computational cost for data division and transmission, *η*|Λ|, is not too large in comparison with the computational cost for discovery, *ζ*|Λ|^2^. The time complexity of using DDCSD to discover signatures from Λ is *O*(|Λ|^2^).

Suppose that the input dataset Λ has a uniform distribution. According to the *γ*-segments included in the *l*-patterns in Λ, the *l*-patterns are assigned to 4^*γ*^*β* (*Υ*,*γ*,*i*,*l*,*d*)-groups. Each of the (*Υ*,*γ*,*i*,*l*,*d*)-groups should contain approximately |Λ|/4^*γ*^ + *κ*|Λ|/4^*γ*^ patterns. In DDCSD, the memory is mainly used to store the patterns in the (*Υ*,*γ*,*i*,*l*,*d*)-groups. The total memory consumption in DDCSD, denoted as, is:

where.

The discovery node handles the discovery routine in DDCSD. If there are too many patterns in the source and candidate datasets, so that they cannot be loaded into memory all at once, the discovery routine will split them into multiple parts and load and process the parts one at a time. In addition, the threshold value for direct discovery, *N*, is decided based on the memory space of discovery nodes so that the patterns in the datasets can be loaded into the memory. Thus, the number of patterns in each of the parts is on the order of *N*. According to the *γ*-segments included in the *l*-patterns in the parts, the *l*-patterns are assigned to (*Υ*,*γ*,*i*,*l*,*d*)-groups. In discovery nodes, the memory is mainly used to store the patterns in the (*Υ*,*γ*,*i*,*l*,*d*)-groups. Based on the above discussion about the total memory consumption in DDCSD, the memory consumption of each discovery node is *τ*|*N*|.

The space complexity of using DDCSD to discover signatures in Λ is *O*(|Λ|). The space complexity of a discovery node is *O*(*N*).

### Performance evaluation

The experimental platform that we used was a cluster of eleven computers, including one main node and ten discovery nodes. The main node was equipped with an Intel Core i7 CPU 870 at 2.93 GHz, 16 GB of memory and 1.5 TB of disk space. Each of the discovery nodes was equipped with an Intel Core i7 CPU 3770 K at 3.50 GHz, 32 GB of memory and 1 TB disk space. The operating system was CentOS release 6.3, and the algorithm tested was coded in JAVA and compiled in JDK 1.6. In this experiment, we used the human whole-genome EST database with 2.46G bases to test the performance of the DDCSD algorithm. In order to avoid impacting the testing, we deleted all remarks and sequences shorter than 36 bases in the database and replaced all universal characters, for example ‘don’t care’, in the sequences with an ‘A’.

When testing the DDCSD algorithm, each recursion only loads the beginning and ending position of the data partition and not the actual data. Only when the discovery needs to happen does it load the data completely into the memory in order to avoid taking up large amounts of memory. In the tests, each *l*-pattern is divided into 2 segments, with the *γ* value set to *l*/2.

In terms of testing the discovery performance of the DDCSD algorithm, we used the human whole-genome EST database as the experimental dataset, ten discovery nodes, and a dataset threshold for direct discovery at 125 MB. The results are shown in Table4. Our data shows that the DDCSD algorithm can discover all signatures under the discovery condition (30,2) from the human whole-genome EST database in 6820 seconds, about 1.89 hours, and discover all signatures under the discovery condition (24,4) from the database in 52366 seconds, about 14.55 hours. When the length of patterns were the same, if the mismatch tolerance *d* is larger, a larger number of strings are compared. Thus, time required is significantly greater than that of when the *d* value is lower. Comparison is difficult when dealing with implementations that were optimized for different tasks. The requirements of discovery algorithms with regard to hardware components, for example the demand on memory size, are different. Although in cases where there is a sufficient amount of memory many existing discovery algorithms, such as Tallymer[32], are able to process the dataset in the experiment. However, the memory requirement of those algorithms is often too large so that they cannot be executed on general-purpose computers with normal memory size. DDCSD uses a divide-and-conquer strategy to recursively divide large datasets into smaller datasets until the split datasets can be processed using the current memory size of discovery nodes. Then, the discovered signature sets from each of the split datasets are integrated to obtain the signature set of the original dataset. Therefore, when the memory size available for the discovery nodes is limited, even to the 32 GB or 16 GB common on regular personal computers or smaller, DDCSD can still process large datasets. The processing ability of DDCSD is not limited by memory size. In addition, by setting the threshold value for direct discovery, DDCSD can limit the memory requirement of discovery nodes during discovery, which ensures that DDCSD can run on a cluster of discovery nodes of different memory sizes.

**Table 4:** **The discovery time for the DDCSD algorithm to discover signatures from the human whole-genome EST database under various discovery conditions**

(***l,d***)	***l*** = 24	***l***= 26	***l***= 28	***l***= 30
*d* = 2	11758	8672	7284	6820
*d* = 4	52366	40384	26100	20093

The experiment uses ten discovery nodes. The time unit is seconds.

**Figure 3:**
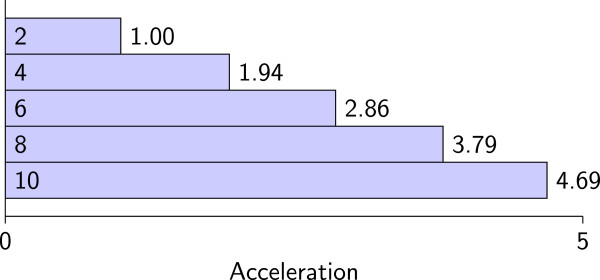
**The acceleration when using various number of discovery nodes.** The values within the inside of the bars are the number of discovery nodes.

In order to test the impact of the number of discovery nodes on the discovery performance of DDCSD, under the discovery condition (24,2), we utilized two to ten discovery nodes to perform signature discovery on the human whole-genome EST database. The dataset threshold for direct discovery is set to 125 M bases. We define acceleration as the ratio of the discovery time when two discovery nodes are used to the discovery time when various number of discovery nodes are used. The acceleration indicates the improvement in discovery performance. The results are shown in Table5 and Figure3. Table5 presents the discovery time for the DDCSD algorithm to discover signatures when various number of discovery nodes were used, and Figure3 presents the acceleration due to the various number of discovery nodes. As we can see, when the number of discovery nodes increases, the discovery time decreases and acceleration increases. For example, when using four discovery nodes, the discovery performance is 1.94 times what it was with two discovery nodes. When using ten discovery nodes, the discovery performance is 4.69 times that of when there was two discovery nodes. The improved discovery performance is linearly related to the number of discovery nodes.

**Table 5:** **The discovery time when various number of discovery nodes were used**

Nodes	2	4	6	8	10
Time	55185	28398	19275	14566	11758

The time unit is seconds.

**Figure 4:**
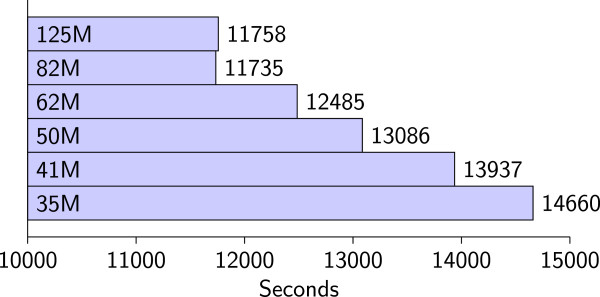
**The discovery time when using various thresholds for direct discovery.** The values within the inside of the bars are the threshold values.

Finally, we tested the effect of thresholds for direct discovery on the discovery performance of DDCSD. This test was done on the human whole-genome EST database using ten discovery nodes with the discovery condition set as (24,2). The threshold value *N* was chosen between 35 and 125 MB. The results are shown in Figure4. From the results, we can see that with the same amount of data, as *N* decreases, discovery time increases, and, discovery time decreases with the increase of *N*. For example, if *N* is set to 35 MB, then discovery time is approximately 24.68% more than that of when *N* is set to 125 MB. The smaller the *N* value, the more recursions are necessary in discovery, and the need for additional computation and overheads in data transmission also increases. However, the memory requirement for discovery processes decreases with the decrease of *N*, as is intuitively obvious. The results also indicate that the size of the database that the DDCSD algorithm can process is not limited by the amount of memory available. As long as a suitable *N* value is set based on the size of the memory in discovery nodes, even when facing large amounts of data, it can still be successfully processed.

## Conclusions

In this research, we proposed a distributed divide-and-conquer-based signature discovery (DDCSD) algorithm. The DDCSD algorithm uses a divide-and-conquer strategy to overcome the problem of processing larger databases, thus solving the disadvantage of previous algorithms that could not process large databases. Also, a parallel computation mechanism on a computer cluster was used to accelerate the signature discovery. Therefore, this algorithm is not limited by the amount of memory available, and can rapidly find signatures in large databases, making it applicable to analysis of NGS and other large amounts of data.
